# Heterogeneous Cytoskeletal Force Distribution Delineates the Onset Ca^2+^ Influx Under Fluid Shear Stress in Astrocytes

**DOI:** 10.3389/fncel.2018.00069

**Published:** 2018-03-16

**Authors:** Mohammad M. Maneshi, Frederick Sachs, Susan Z. Hua

**Affiliations:** ^1^Department of Physiology and Biophysics, University at Buffalo, Buffalo, NY, United States; ^2^Department of Mechanical and Aerospace Engineering, University at Buffalo, Buffalo, NY, United States

**Keywords:** astrocytes, cytoskeletal forces, traumatic brain injury (TBI), fluid shear stress, FRET, mechanosensitive ion channel (MSC)

## Abstract

Mechanical perturbations increase intracellular Ca^2+^ in cells, but the coupling of mechanical forces to the Ca^2+^ influx is not well understood. We used a microfluidic chamber driven with a high-speed pressure servo to generate defined fluid shear stress to cultured astrocytes, and simultaneously measured cytoskeletal forces using a force sensitive actinin optical sensor and intracellular Ca^2+^. Fluid shear generated non-uniform forces in actinin that critically depended on the stimulus rise time emphasizing the presence of viscoelasticity in the activating sequence. A short (ms) shear pulse with fast rise time (2 ms) produced an immediate increase in actinin tension at the upstream end of the cell with minimal changes at the downstream end. The onset of Ca^2+^ rise began at highly strained areas. In contrast to stimulus steps, slow ramp stimuli produced uniform forces throughout the cells and only a small Ca^2+^ response. The heterogeneity of force distribution is exaggerated in cells having fewer stress fibers and lower pre-tension in actinin. Disruption of cytoskeleton with cytochalasin-D (Cyt-D) eliminated force gradients, and in those cells Ca^2+^ elevation started from the soma. Thus, Ca^2+^ influx with a mechanical stimulus depends on local stress within the cell and that is time dependent due to viscoelastic mechanics.

## Introduction

During traumatic brain injury (TBI), brain cells experience transient shear stresses that alter the forces in structural proteins (Hemphill et al., 2015). Universally, cells subjected to shear forces show an elevation of intracellular Ca^2+^ (Tzima et al., 2005; Ravin et al., 2012; Li et al., 2014). The Ca^2+^ response to mechanical stimuli is highly nonlinear so the response to a stimulus depends critically on the stimulus waveform (Cullen et al., 2011; Maneshi et al., 2015). Rapid shear deformations increase the membrane permeability to Ca^2+^ and small dye molecules and decreased cell viability (LaPlaca et al., 2005; Cullen et al., 2011). The response depends not only on the magnitude of the stimulus but also on the rate of loading and the repetition rate (LaPlaca et al., 2005; Cullen et al., 2011; Maneshi et al., 2015). These studies suggest that local forces are not uniform in space or time, and there is a viscoelastic or poroelastic coupling of body stress to the Ca^2+^ influx (Sachs and Sivaselvan, 2015).

The initial increase of Ca^2+^ could be the results of Ca^2+^ entry through mechanosensitive ion channels (MSCs), a release from the endoplasmic reticulum (ER) stores (Verkhratsky and Shmigol, 1996; Dunn et al., 2013) or activation of inositol trisphosphate (IP_3_) pathways (Parri and Crunelli, 2003; Fiacco and McCarthy, 2006). Using primary astrocytes as model, we have shown that a Ca^2+^ *influx* is essential for the Ca^2+^ rise under shear stress (Maneshi et al., 2015). In astrocytes the dominant Ca^2+^ influx pathways are mechanosensitive N-methyl-D-aspartate receptors (NMDARs; Maneshi et al., 2017). Since these channels are connected to cytoskeleton via binding proteins such as α-actinin (Wyszynski et al., 1997; Husi et al., 2000), stretching the cytoskeleton by fluid drag on the cell can increase NMDAR gating. The release of Ca^2+^ from ER stores contributes ~50% of total Ca^2+^ response probably through Ca^2+^-induced Ca^2+^ release (CICR) that is slower than the rate of influx (Maneshi et al., 2015). Several recent studies using brain slices have shown that Ca^2+^ transients at the distal processes of astrocyte have different kinetics than those in the somata (Lind et al., 2013; Kanemaru et al., 2014; Srinivasan et al., 2015). While Ca^2+^ rise in the processes depends on both Ca^2+^ entry and Ca^2+^ release, those in the soma depend mainly on release (Srinivasan et al., 2015). Thus, shear stimuli may activate different Ca^2+^ signaling pathways depending on the spatiotemporal distribution of forces. Delineating the early Ca^2+^ responses is an important step in understanding the effectors that lead to pathology.

Microfluidic chips combined with optical probes enable delivering precisely controlled fluid shear stress to live cells and simultaneously measuring optical signals in real-time. This technique has been used to analyze many cellular signals and biochemical events in live cells (de Campos et al., 2015; Li et al., 2016; Fresta et al., 2017). However, no study has yet directly linked the cytoskeletal forces to Ca^2+^ influx during TBI.

Here we used tissue cultured adult primary rat astrocytes in a microfluidic chamber driven by a fast pressure servo to generate well-defined fluid shear. We simultaneously measured the protein forces and the Ca^2+^ responses in cells co-expressing the FRET based force sensors actinin-cpstFRET (Meng and Sachs, 2012; Guo et al., 2014) and the genetically encoded Ca^2+^ probe jRCaMP1a (Dana et al., 2016). We show that rapid stimuli generate tension gradients in cells with the highest tension towards the upstream edge. Thus, mechanically induced Ca^2+^ dynamics is governed by force gradients and these vary in time and space.

## Materials and Methods

### Flow Chamber and Shear Stress Generation

The microfluidic flow chambers were made of parallel glass slides with Polydimethylsiloxane (PDMS) walls (Rahimzadeh et al., 2011; Maneshi et al., 2015). The flow chamber was 1000 μm wide and 100 μm in height. A fast pressure servo generated defined waveforms with a time resolution of ~1 ms (Besch et al., 2002; Maneshi et al., 2015). The shear stress in the chamber and the rise time was calibrated using microbeads and volume collection methods as previously described (Maneshi et al., 2015). The microfluidic chamber’s glass floors were coated with human fibronectin (BD Bioscience) to enhance cell growth.

### Cell Culture and Transfection

Primary adult astrocytes obtained using gelatin-sponge implants from adult Sprague-Dawley rat brains (Langan et al., 1995) were provided by Dr. Thomas Langan (University at Buffalo) at passage number 2–3. Cells were maintained in DMEM, with 10% fetal bovine serum (FBS) and 1% Penicillin/Streptomycin, and used in experiments between passages 3 and 10. Cells were transferred to the microfluidic chambers when cells in the culture flasks reached 95% confluence, and they were cultured in the chamber for 2–3 days until reaching 50% confluence. Cells were then transfected with actinin-cpst-FRET probes or co-transferred with actinin-cpst-FRET and jRCaMP1a, and cultured for another 48 h. Media in the chamber was changed every 24 h and changed 6 h before experiments.

### FRET Measurements

To measure the forces in cytoskeletal proteins, a force sensitive FRET probe cpst-FRET (Meng and Sachs, 2012; Guo et al., 2014) was genetically incorporated into host protein α-actinin (actinin-cpst-FRET). The probe consists a FRET pair of Cerulean (CFP) and Venus (YFP) bound with short flexible linker (Meng and Sachs, 2012; Guo et al., 2014). Mechanical forces applied to the probe twist the dipoles from parallel to diagonal decreasing FRET efficiency. The force sensitivity of the probes was calibrated in solution using DNA springs according to a previously described method (Tseng et al., 2009; Meng and Sachs, 2012). The cpst-FRET probes are sensitive to forces <10 pN (Meng and Sachs, 2012). The CFP and YFP images were acquired simultaneously using a quad-view imaging system. The two images were aligned and the background was subtracted using ImageJ. The FRET accepter/donor ratio was calculated for each ROI, and an inverted FRET ratio was used to represent the forces, consistent with our previous publications (Guo et al., 2014).

### Ca^2+^ Measurements

Intracellular Ca^2+^ was measured using two probes, the genetically encoded calcium indicator jRCaMP1a (Plasmid #61562, Addgene, Cambridge, MA, USA), and Rhod-2 (Invitrogen). jRCaMP1a was transfected using Effectene Transfection Reagent 48 h prior to experiments. The dye was dissolved in DMSO and diluted to a final concentration of 5 μM in saline.

### Fluorescence Imaging

The fluorescence signals were measured using a multichannel LED light source (XLED1, X-Cite) with excitation filters (430–465 nm) for FRET and (540–600 nm) for Ca^2+^. The emission lights were separated using a quad-view imaging system (QV2 four-channel imager, Photometrics, AZ, USA) with filter sets, 482/25 nm, 544/25 nm and 641/75 nm for CFP, YFP and RFP channels, respectively, and a triple band dichroic mirror (FF459/526/596-Di01, Semrock). The images were acquired using an EM-CCD camera (EM C9100-13C, Hamamatsu, Japan) cooled to −85°C to minimize dark noise.

### Membrane Tension Measurements

A molecular rotor, 2-carboxy-2-cyanovinyl-julolidine farnesyl ester (FCVJ), was used to measure the tension in the lipid bilayer (Haidekker et al., 2001). FCVJ probe was a gift from Dr. Mark Haidekker (University of Georgia). The rotor loses energy at its excited state when free spinning in the membrane and the intensity of the emission is inversely proportional to free volume of the bilayer. The probes were dissolved in DMSO, aliquoted, and immediately frozen. Prior to experiments, aliquot (20 μl at 10 mM) was added to 400 μl of Gibco FBS (Thermo Fisher Scientific, Grand Island, NY, USA) and vigorously vortexed for 10 s. The solution was loaded and the cells were incubated for 30 min.

### Solutions and Reagents

Normal saline containing 1 mM CaCl_2_ was the control solution. Cytochalasin-D (Cyt-D; Sigma, St. Louis, MO, USA) was dissolved in DMSO to a 10 mM stock solution kept frozen, and diluted to a final concentration of 10 μM. Jasplakinolide (J7473, Thermo Fisher Sciences) was dissolved in DMSO to 1 mM stock solution and diluted to 500 nM before experiments.

### Data Analysis

Relative Ca^2+^ intensity was calculated using

$$\frac{\Delta F}{F_{0}}=\frac{F-F_{0}}{F_{0}},$$

where *F* and *F*_0_ are the mean fluorescence intensities of individual cells at time *t* and *t* = 0, respectively. The mean calcium changes were an average over *N* selected cells from each image and from more than four experiments for each condition. FCVJ intensity was calculated using the similar method, where mean intensity changes were calculated after background subtraction. We used a new cell culture for each experiment. The data is shown as mean ± standard error of the mean (SEM). Statistical analysis used a two-sample *t*-test. A value of *p* < 0.05 was considered statistically significant.

## Results

### Rapid Shear Stimuli Generate Non-uniform Protein Forces in Single Cells

**Figure 1:**
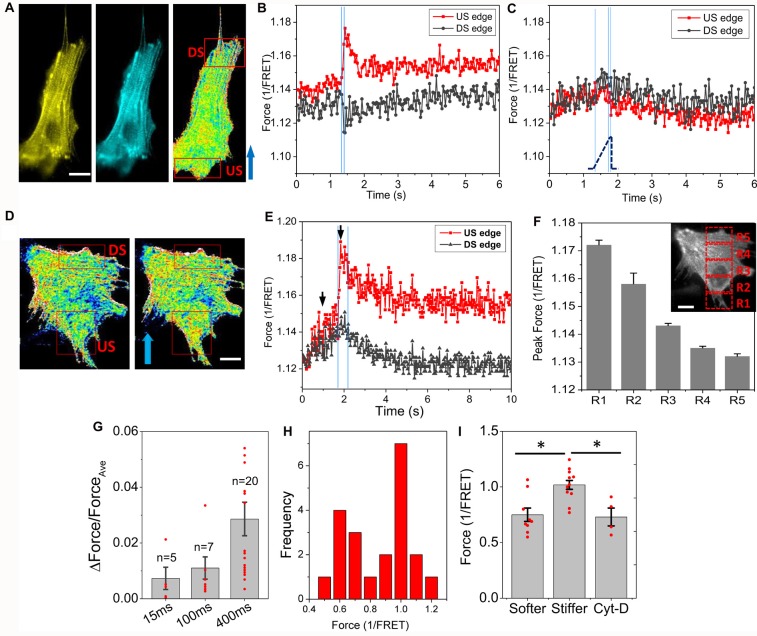
Rapid fluid shear pulse generates non-uniform force distribution in astrocytes.** (A)** Fluorescent images (YFP, CFP) and inverse FRET ratio of an astrocyte expressing actinin-cpstFRET. **(B)** Changes of actinin force in two ROIs marked in **(A)** in response to a narrow shear pulse (23 dyn/cm^2^, 15 ms, 2 ms rise time), showing narrow shear pulse produced tension in actinin at the upstream edge (US) and compression at downstream edge (DS). Blue arrow indicates flow direction. **(C)** Force response to a slow rise shear pulse (400 ms rise time) with same amplitude did not produce force gradient. The blue lines indicates the on, saturation and off of the pulse. **(D,E)** Force map (inverse FRET ratio, **D**) before and within the pulse at the times indicated by black arrows in **(E)** with a broader pulse (23 dyn/cm^2^, 400 ms, 2 ms rise time). Red color indicates higher tension, and blue color, lower tension. Notice the fast increase and slow recovery of force in actinin in upstream region following an effectively instantaneous step pulse. **(F)** Actinin forces measured from the selected windows (insert in **F**) at the end of the pulse showing a gradient along flow direction. **(G)** Normalized force difference between upstream and downstream edges for different pulse width showing most cells saturated at 400 ms pulse width.** (H)** Histogram of mean actinin forces in individual cells at its resting stage showing a bimodal distribution.** (I)** Mean actinin force (1/FRET ratio) of resting cells shows the responsive cells (~40% cells) have lower tension at resting state (softer and stiffer cells, *n* = 9; Cytochalasin-D (Cyt-D) cells, *n* = 4, the comparison with Cyt-D cells was based on *n* = 4). *p < 0.005. Scale bars indicate 10 μm.

A narrow shear stress pulse (23 dyn/cm^2^, 2 ms rise time, 15 ms duration) caused a significant Ca^2+^ rise in astrocytes, but the response was non-uniform in time and space in single cells. To determine whether the stimulus generated heterogeneous forces in the cell, we measured the force distribution with actinin-cpst-FRET, and found that a rapid fluid shear caused non-uniform change in actinin forces (Figures 1A,B). We observed increased tension at the upstream edges of the cells (US edge), and decreased tension at the downstream edge (DS edge, Figure 1B). In contrast, a slow shear stress ramps to the same amplitude caused a minimal and rather uniform increase in actinin stress across the cell (Figure 1C). Using a broader square pulse (400 ms) we found that the protein forces reached a maximum in ~100 ms (Figures 1D,E), while some cells showed a secondary increase reaching a summit at ~400 ms possibly due to the breakdown of some crosslinking proteins (Figures 1E,G). The forces above the Ca^2+^ thresholds did not disrupt cell adhesion. The spatial and temporal distribution of forces showed a gradient along flow direction (Figure 1F). The average strain of the cells at the end of the pulse was only ~4%. A histogram of actinin forces of individual cells in rest showed a clear bimodal distribution (Figure 1H). Cells with fewer stress fibers had lower actinin tension that was comparable to Cyt-D treated cells (Figure 1I, *p* < 0.005). Those cells (~40% of cell population) responded to fluid shear stress with significant force gradients. The shear-induced gradient was smaller in cells that had abundant actin bundles. Using a finite element method, we modeled a viscoelastic cell body under the fluid shear stress, and the results showed a tension distribution maximal at the upstream edge and with reduced strain at the downstream edge in softer cells (see Supplementary Figure S1). It is consistent with our experimental data. Thus, an averaged impact force cannot be used to assess the activation of cell processes; the force at the high stress points can be many fold higher than the average.

### Initial Ca^2+^ Rise Is Strictly Governed by the Cytoskeletal Force Gradient

**Figure 2:**
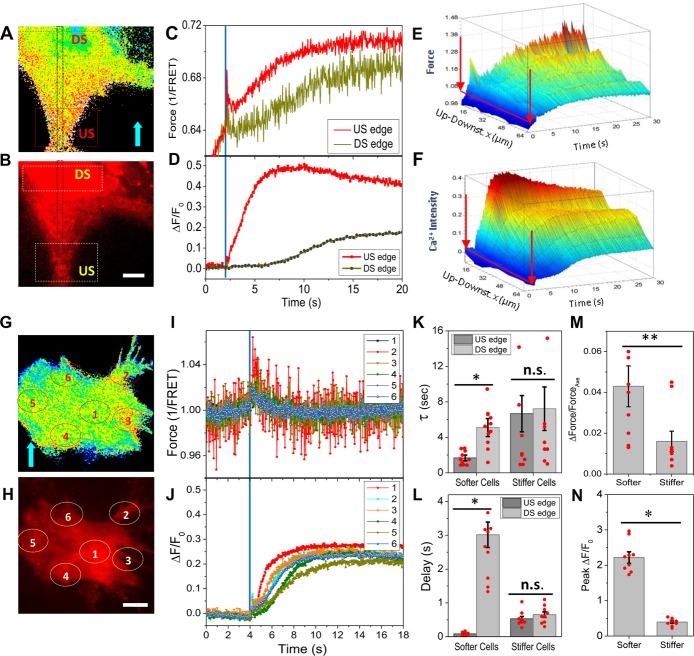
Simultaneously measured forces and intracellular Ca^2+^ in response to shear pulses.** (A,B)** Typical force map generated with actinin-cpstFRET probes and Ca^2+^ with Rhd-2 in a soft cell. **(C,D)** Changes of forces and Ca^2+^ in response to a rapid shear pulse (23 dyn/cm^2^, 400 ms, indicated by blue lines), showing Ca^2+^ rise began at the upstream end of the cell where there was higher force. **(E,F)** 3D map of evolution of forces and Ca^2+^ along a strip (gray window in **A,B**), showing the Ca^2+^ propagating as a wave from upstream to downstream of the cell. **(G–J)** Typical force **(G,I)** and Ca^2+^ responses **(H,J)** to the same shear pulse in a stiffer cell, showing a small uniform transient change in force and small Ca^2+^ rise. **(K,L)** The Ca^2+^ increase is faster with little latency at the upstream edge compared with downstream edge in the softer cells, but it was slower with delay in stiffer cells (*n* = 11, softer cells; *n* = 9, stiffer cells). *p < 0.005. **(M,N)** Statistical analysis shows the force gradients and peak Ca^2+^ are higher in the softer cells. **p < 0.05. Error bars indicate SEM. Scale bars indicate 10 μm.

To correlate the Ca^2+^ rise with the local protein forces, we measured the distribution of forces and the changes in Ca^2+^ simultaneously by co-expressing Ca^2+^ probes with the force sensor. A rapid shear pulse caused a Ca^2+^ rise starting at the upstream end of the cell where there also was high stress (Figures 2A,B). The Ca^2+^ peaked much faster (~4 s) at the upstream edge and peaked in ~10 s in the center of cell body (DS edge, Figures 2C,D). A 3D plot of time evolution of forces and Ca^2+^ along the center band (Gray window in Figures 2A,B) shows a transient increase in force at the upstream edge that correlated with a fast increase in Ca^2+^, and the Ca^2+^ subsequently propagated to the soma as a wave (Figures 2E,F). The time constant of Ca^2+^ rise at the upstream edge is ~3 times faster than the soma possibly due to the confined volume of the narrow part of the cell and the increases in Ca^2+^ concentration drive CICR (Figure 2K, softer cells, *p* = 0.0001, *n* = 11). This leads to a ~3 s delay of peak Ca^2+^ between the upstream edge and the soma (Figure 2L, softer cells, *p* = 0.0001, *n* = 11). In stiffer cells having abundant actin bundles, a more uniform transient forces appeared over the entire cell (Figures 2G,I), and the Ca^2+^ started randomly and increased to a lesser extent (Figures 2H,J). There was no observable force gradient in these cells (Figure 2M, *p* = 0.019, *n* = 9). The Ca^2+^ increased slower than the softer cells (Figure 2K, stiffer cells) and with a 500 ms delay (Figure 2L, stiffer cells). The peak Ca^2+^ is much lower in the stiffer cells (Figure 2N, *p* = 0.0001, *n* = 9). These data indicate that force sensitive Ca^2+^ influx occurred predominantly at the front edge of the cell.

### Viscoelasticity of Cytoskeleton Is Responsible for Nonlinear Ca^2+^ Responses

**Figure 3:**
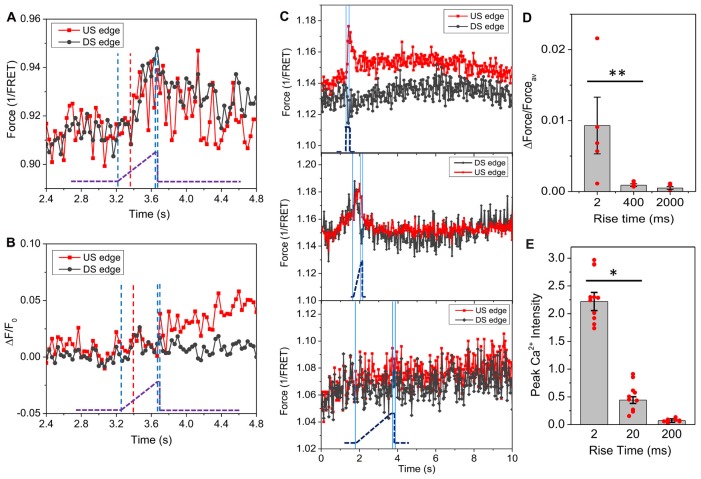
Effect of stimulus features on distribution of actinin force in single cells.** (A)** Time course of actinin forces in response to a slow ramping shear pulse (23 dyn/cm^2^, 15 ms, 400 ms rise time), showing an even increase in force with 170 ms delay (indicated by red dashed line). The blue lines indicates the on, saturation and off of the pulse. The pronounced latency suggests actinin tension is viscoelastically driven from an early responding component. **(B)** Simultaneously measured Ca^2+^ response showing minimal Ca^2+^ rise at the upstream edge.** (C)** Same measurements with rise times of 2400 and 2000 ms, respectively, showing the force gradient was only generated below 400 ms. **(D,E)** Comparison of force gradients with peak Ca^2+^ values for stimuli (23 dyn/cm^2^, 15 ms) with different rise times, showing high Ca^2+^ response to the fast pulse is consistent with high force gradient (for forces, 2 and 400 ms: *n* = 6; 2 s: *n* = 4; For Ca^2+^: *n* = 30). *p < 0.005; **p < 0.05.

If cytoskeletal force is a direct input to Ca^2+^ rise, due to viscoelasticity the distribution of the forces should be sensitive to the stimulus rise time. Slow ramps (400 ms) to the same amplitude did not produce force gradients but generated delayed and uniform tension throughout the cells (Figure 3A). Interestingly, there was no change in actinin force until 170 ms after the onset of the ramp (indicated by red dashed line, Figure 3A), at which the estimated stimulus reached 11 dyn/cm^2^, the steady state threshold for a Ca^2+^ response. Ca^2+^ increased slightly at upstream edge after the shear ramped to its maximum at 400 ms (Figure 3B). The force sensitivity to the rise time sets a limit of ~100 ms to reach the maximum local strain (Figure 3C). We compared the force gradient and Ca^2+^ peak intensity for different rise times and found that higher force gradients result in higher Ca^2+^ peaks (Figures 3D,E, for Figure 3D, *p* = 0.047, *n* = 6; for Figure 3E, *p* = 0.003, *n* = 30). It is the local force gradients rather than the average force that is critical to the Ca^2+^ influx. This result explains why some research groups did and did not observe the effect of strain rate on Ca^2+^ rise in different TBI models (Cater et al., 2006).

**Figure 4:**
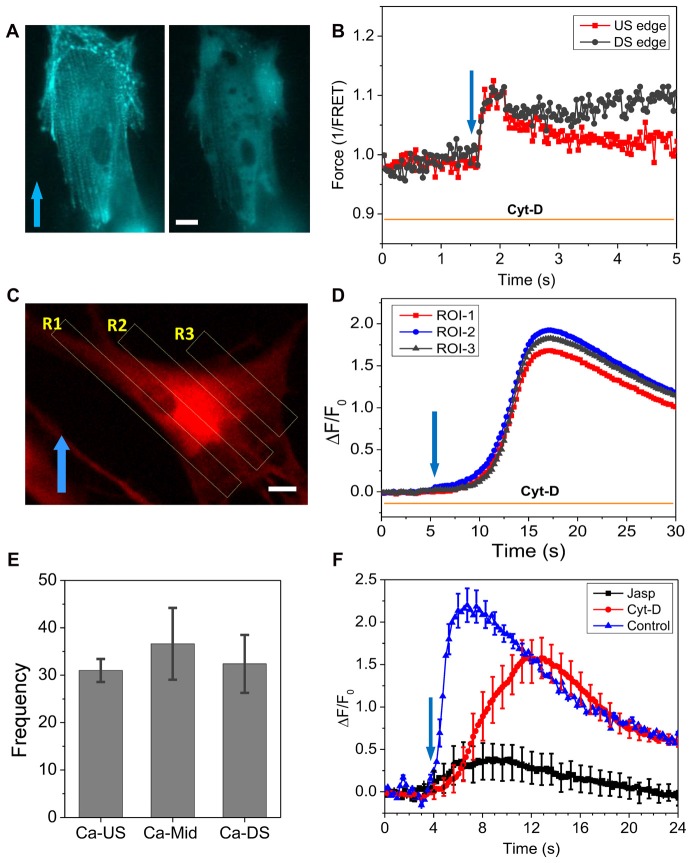
Effect of Cyt-D on force distribution and Ca^2+^ influx. **(A)** Fluorescence image of actinin-cpst-FRET (YFP channel) of an astrocyte cell before and after treatment of 10 μM Cyt-D, showing the drug disrupted actin. **(B)** Force responses in upstream (red) and downstream regions (black) to a rapid shear pulse (23 dyn/cm^2^, 15 ms, 2 ms rise time), showing disruption of F-actin eliminated the force gradient. **(C,D)** Ca^2+^ responses in three ROIs marked in **(C)**, showing the drug treatment produced a uniform but delayed Ca^2+^ rise in the cell. **(E)** Frequency of Ca^2+^ response starting from different regions showing a high probability at the cell soma in drug treated cells (total 72 cells from four runs). **(F)** Comparison of Ca^2+^ responses to a shear pulse in Cyt-D and jasplakinolide treated cells, showing Cyt-D eliminated the fast Ca^2+^ response while Jasplakinolide largely reduced the peak (*n* = 30 for each curve). Cytoskeleton is critical to the Ca^2+^ response. Scale bars indicate 10 μm.

To further assess the roles of cytoskeleton, we modified F-actin with Cyt-D (10 μM) that disrupted most of stress fibers (Figure 4A). After Cyt-D treatment, a rapid shear pulse (23 dyn/cm^2^, 15 ms, 2 ms rise time) was not able to produce force gradient in actinin and actinin force was quite uniform throughout the cell (Figure 4B). This indicates that the non-uniform distribution of forces in cells is the result of a linked cytoskeleton. The Ca^2+^ influx was not inhibited by the Cyt-D, although the elevation happened much slower than in controls (peaked in ~4 s, Figure 2), the Ca^2+^ peaked in ~11 s (Figure 4D). There was no longer preference for the Ca^2+^ rise to occur at the upstream edge of the cell (Figures 4C,D). The statistical analysis of 72 Cyt-D treated cells from four experiments shows that Ca^2+^ elevation starts from the soma in most cells (Figure 4E). As controls, we treated cells with jasplakinolide that enhances actin polymerization (Bubb et al., 2000). The drug reduced the Ca^2+^ peak by ~80% (Figure 4F), becoming similar to the behavior of stiffer cells (Figures 2G–J).

## Discussion

A transient fluid shear stress generates Ca^2+^ influx in astrocytes, and the response depends critically on the features of the stimulus (Maneshi et al., 2015). We mapped the forces in cytoskeletal linking proteins and intracellular Ca^2+^ activity in time and space in cells subjected to fluid shear stress, and showed that a uniform shear stress can generate heterogeneous cytoskeletal forces, and the onset of Ca^2+^ influx correlates to the force gradients. Higher local force triggers the initial Ca^2+^ influx that typically occurs at the upstream edge of the cells. This force gradient that was only produced with a fast stimulus with a rise time below ~100 ms, the relaxation time of the viscoelastic cell body (Maneshi et al., 2015), is the basis of the nonlinear Ca^2+^ response to shear stimuli. Fluid shear stress causes a minimal deformation (~4%) of cells, but it is above the threshold for Ca^2+^ response (Maneshi et al., 2015). This study shows that the local forces could be an order of magnitude higher than the average and the force sensitive pathways are only activated at those locations that have high cytoskeletal forces.

The heterogeneous response is determined by the intrinsic mechanical properties of the cells in their resting state. Softer cells that have fewer stress fibers and lower tension in the resting state showed stress gradients, while in cells with abundant stress fibers and higher pre-tension, we observed spatially uniform stress transients (Figure 1). Stress fibers are distributing stresses throughout the cell. Finite element analysis of a viscoelastic cell models (Supplementary Figure S1) showed that rapid narrow shear stimuli produce a tension dominant domain at the upstream edge and compressive domain at the downstream edge of the cell, and the effect was diminished in stiffer cells. Modification of the cytoskeleton with Cyt-D eliminated stress gradient in cells and abolished the initial Ca^2+^ influx (Figure 4). This evidence indicates that the cytoskeleton structure and its pre-stress states are the determinants of the cell mechanical response and thus the initial influx.

We previously reported that at least two types of MSCs contribute to force-induced influx in astrocytes but the primary source are force sensitive NMDARs (Maneshi et al., 2017). Many MSCs, including NMDARs, link with the actin cytoskeleton via crosslinking proteins such as actinin (Wyszynski et al., 1997). These channels could be responsible to the initial influx seen at high stressed regions. However, the rise in Ca^2+^ is far delayed from the termination of the stimulus raising the question of what cellular components provide the “memory” of the earlier stimulus (Figure 2). We suggest that the initial mechanical stress breaks some cytoskeletal links and as they relax, stress is transferred to the components that drive the Ca^2+^ permeant MSCs. The highly delayed Ca^2+^ involves other mechanisms such as CICR that do not directly operate by local forces (Verkhratsky and Shmigol, 1996; Dunn et al., 2013).

The coupling of fluid shear stress to MSCs is clearly not direct evidence by the pronounced latency. Body stresses due to disrupted cytoskeletal links can be the coupling (Orr et al., 2006; Hayakawa et al., 2008). Alternatively, shear stress causes deformation and flow in the lipid bilayer where most publications link channel activation to bilayer tension. Using molecular rotors to measure changes of lipid membrane fluidity or free volume under shear stress (Haidekker et al., 2001), we found that similar tension gradients in the lipids occurred with shear stress (Supplementary Figure S2), consistent with the previous report on endothelial cells (Butler et al., 2002). The bilayer tension increased at the upstream edge and decreased at the downstream edge, but it returned to the resting state immediately after the shear pulse, in contrast to actinin tension that took more time to relax (Supplementary Figure S2). Interestingly, the release of bilayer tension at the downstream edge occurred faster than the increase of tension at the upstream edge, while actin disruption with Cyt-D significantly increased the recovery time. This supports the coupling of cytoskeletal forces to bilayer tension that can activate the channels (Akinlaja and Sachs, 1998). In conclusion, the cytoskeleton plays an import role in cell response to shear stimuli, it produces high forces in specific regions of a cell while retaining the shape and volume of the bilayer membrane when it is deformed. The observed Ca^2+^ dynamics under shear stress are governed by the viscoelastic properties of the cytoskeleton and the bilayer.

## Author Contributions

MMM designed and performed the experiments and wrote the manuscript. FS interpreted data and wrote the manuscript. SZH designed the experiments, analyzed and interpreted data and wrote the manuscript.

## Conflict of Interest Statement

The authors declare that the research was conducted in the absence of any commercial or financial relationships that could be construed as a potential conflict of interest.
