# Supplementary material for: Heterogeneous Cytoskeletal Force Distribution Delineates the Onset Ca2+ Influx Under Fluid Shear Stress in Astrocytes
Source: Front Cell Neurosci. 2018 Mar 16;12:69. doi: 10.3389/fncel.2018.00069 (PMC5864927; doi:10.3389/fncel.2018.00069)
Supplement: Supplementary file 1 [file Presentation_1.pdf]

Supplementary Materials:

Supplementary Materials SM 1. FEA model of stress distribution in cells under fluid shear stress.

We used viscoelastic cell model to calculate the stress distribution in cells subjected to narrow shear stimulus used in the experiments. The geometry was generated in the ANSYS Design Modeler. The height of perfusion channel was 100 µm with a length of 1.5 cm to replicate the microfluidic chamber used experimentally. The fluid in the model was water. A pressure pulse (2 ms rise time, 8 ms duration and 2 ms fall time) applied at the channel entrance mimicked the pressure generated by the servo pump. The pressure distribution at the interface of the fluid-solid was then imported to deformable mechanical model of the cell.

To model the viscoelastic hyperelastic behavior of the soft cell we used a first order Ogden model with a Prony shear relaxation of 0.1 s:

$$\psi = \frac{\mu}{2}(\overline{I_1} - 3) + \frac{1}{d}(J - 1)^2$$

where $\overline{I_1}$ is the deviatoric first principal invariant, J is the Jacobian, μ is the initial shear modulus and d is the incompressibility parameter. The Prony shear moduli was calculated by:

$$G(t) = G(\infty) + G_1 \exp(-\frac{t}{0.1})$$

The model parameters were:

$$\rho = 1000\frac{kg}{m^3}, \mu = 1038\, pa, A_1 = 2.76, d = 0.0001$$

To model the elastic behavior of a stiffer cell we used an isotropic linear elastic model with the parameters below:

$$\rho = 1000\frac{kg}{m^3}, E = 5000\, pa, v = 0.489$$

Figure S1 shows the von-Mises (equivalent) stress distribution in a cell body with the fluid flow from left to right. The transient pressure pulse produced higher tension at the upstream end of the softer cell, while it reduced the tension at the downstream end of the cell (Supplementary Figure S1a). It decreased the stress in the stiffer cell uniformly (Supplementary Figure S1b). This is consistent with the experimental results showing distribution of cytoskeletal stresses depends on the viscoelastic properties of the cell.

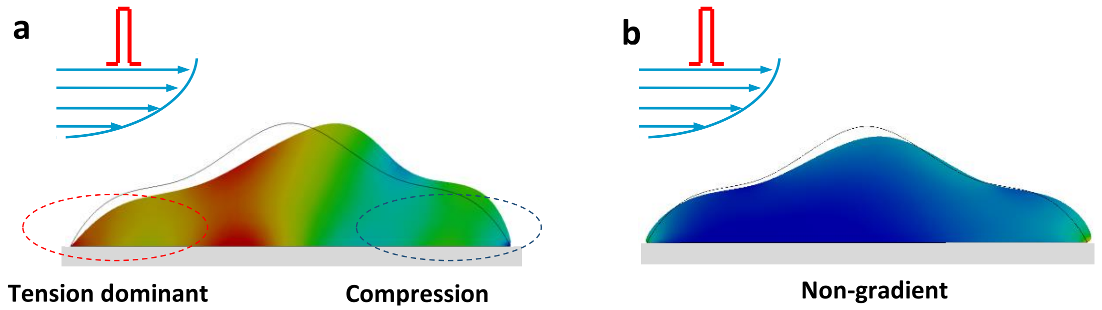

Figure S1. FEA model of a viscoelastic cells under fluid shear stress. a, b Stress distribution of a softer cell (a) and a stiffer cell (b) under fluid shear stress generated by pressure pulse, showing a tension dominant region at the upstream end and a compression dominant region at the downstream end in softer cell (a) while minimal gradient in the stiffer cell (b). The shear stress was applied at t = 0 and the stress distribution was calculated at the end of the puls**e.**

Supplementary Materials SM 2. Membrane tension measured using FCVJ molecular rotors. The bilayer tension was measured using lipid soluble probes molecular rotor FCVJ whose response depends upon the local viscosity. An increase in bilayer tension increases the free volume of the membrane and lowers viscosity for the probe. A square shear pulse (23 dyn/cm^2, 400 ms) applied to cells labelled with FCVJ, showed a rapid increase in bilayer tension at the upstream edge of the cell and a decrease in tension at the downstream edge (Supplementary Figure S2). This resulted in a tension gradient in the bilayer of the cell. Both tension and compression recovered to the initial state within ~50 ms post pulse. Since MSCs tend to activate with increasing bilayer tension (Kloda et al. 2007), the open probability of the channel will be a transient at the onset of flow.

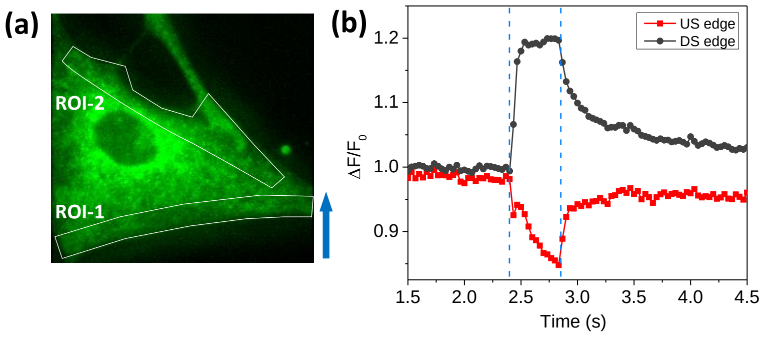

Figure S2. Change of membrane tension under a shear pulse. Square shear pulse 23 dyn/cm^2, 400 ms was applied between the dashed lines. Changes in tension at upstream edge (red curve, from ROI-1 indicated in (a)) and downstream edge (black curve, from ROI-2 in (a)), showing a shear pulse generated non-uniform distribution of tension in the membrane.

Kloda A, Lua L, Hall R, Adams DJ, Martinac B. 2007. Liposome reconstitution and modulation of recombinant N-methyl-D-aspartate receptor channels by membrane stretch. Proceedings of the National Academy of Sciences of the United States of America. Jan 30;104:1540-1545.
